# The role of Cx36 and Cx43 in 4‐aminopyridine‐induced rhythmic activity in the spinal nociceptive dorsal horn: an electrophysiological study in vitro

**DOI:** 10.14814/phy2.12852

**Published:** 2016-07-26

**Authors:** Christopher W. P. Kay, Daniel Ursu, Emanuele Sher, Anne E. King

**Affiliations:** ^1^ School of Biomedical SciencesUniversity of LeedsLeedsLS2 9JTUnited Kingdom; ^2^ Eli Lilly & Co., Lilly Research CentreErl Wood ManorWindleshamSurreyGU20 6PHUnited Kingdom

**Keywords:** Connexins, pain, rhythmic firing, substantia gelatinosa

## Abstract

Connexin (Cx) proteins and gap junctions support the formation of neuronal and glial syncytia that are linked to different forms of rhythmic firing and oscillatory activity in the CNS. In this study, quantitative reverse transcription polymerase chain reaction (RT‐qPCR) was used to profile developmental expression of two specific Cx proteins, namely glial Cx43 and neuronal Cx36, in postnatal lumbar spinal cord aged 4, 7, and 14 days. Extracellular electrophysiology was used to determine the contribution of Cx36 and Cx43 to a previously described form of 4‐aminopyridine (4‐AP)‐induced 4–12 Hz rhythmic activity within substantia gelatinosa (SG) of rat neonatal dorsal horn (DH) in vitro. The involvement of Cx36 and Cx43 was probed pharmacologically using quinine, a specific uncoupler of Cx36 and the mimetic peptide blocker Gap 26 which targets Cx43. After establishment of 4–12 Hz rhythmic activity by 4‐AP (25 *μ*mol/L), coapplication of quinine (250 *μ*mol/L) reduced 4‐AP‐induced 4–12 Hz rhythmic activity (*P <* 0.05). Preincubation of spinal cord slices with Gap 26 (100 *μ*mol/L), compromised the level of 4‐AP‐induced 4–12 Hz rhythmic activity in comparison with control slices preincubated in ACSF alone (*P <* 0.05). Conversely, the nonselective gap junction “opener” trimethylamine (TMA) enhanced 4–12 Hz rhythmic behavior (*P <* 0.05), further supporting a role for Cx proteins and gap junctions. These data have defined a physiological role for Cx36 and Cx43 in rhythmic firing in SG, a key nociceptive processing area of DH. The significance of these data in the context of pain and Cx proteins as a future analgesic drug target requires further study.

## Introduction

Gap junctions support electrical signaling and permit rapid intercellular exchange of small molecules including ions, second messengers, nutrients, and metabolites (Sohl et al. 2005). Gap junction channels consist of a pair of hemichannels that connect opposing cells, either neurons or glia, through connexin (Cx) protein subunits (Theis et al. 2005). Identification and characterization of a family of 20 Cx genes has given an improved understanding of their roles in the immature and adult central nervous system (CNS), highlighting diverse functions ranging from formation of neuron‐glial syncytia to facilitation of rhythmic neuronal firing or distributed network synchrony (Sohl et al. 2004). In spinal cord, expression of neuronal Cx proteins such as Cx36 and Cx45 (Chapman et al. 2013; Condorelli et al. 2000) and of glial subtypes including Cx43 and Cx32 (Rash et al. 2001; Lee et al. 2005) is established.

The presence of heterogeneous Cx subtypes in spinal cord dorsal and ventral horn raises the question of their potential physiological significance and whether gap junction connectivity could influence sensory or motor functions. For the latter, a role for gap junctions in coordinating and generating motoneuron output is proposed, especially in the immature spinal cord (Kiehn and Tresch 2002) and mixed chemical–electrical synapses that express, for example, Cx36 have been described (Bautista et al. 2014). For spinal somatosensory systems, attention has focused largely on the possible contributions of gap junctions to modulation of nociceptive processing or pain facilitation. By virtue of their ability to facilitate intercellular communication and spreading excitation, astroglial gap junctions and Cx proteins are implicated in pathological pain, mirror pain, and mechanisms of central sensitization (Spataro et al. 2004; Wieseler‐Frank et al. 2004). Animal models of inflammatory pain or nerve injury‐induced chronic pain indicate a dynamic plasticity in the function of gap junctions and expression of Cx proteins that may drive pathological alterations (Wu et al. 2012). Furthermore, gap junction or hemichannel conductance is not simply a passive phenomenon but is also subject to active modulation by a variety of endogenous factors including, for example, proinflammatory molecules (Orellana et al. 2013).

Previously, we characterized a novel form of rhythmic activity that is manifested within nociceptive circuitry of neonatal rat substantia gelatinosa (SG) in vitro and is reliant on the chemical and electrical neurotransmission (Asghar et al. 2005). Subsequently, we used a 4‐aminopyridine (4‐AP) model of spinal dorsal horn (DH) hyperexcitability (Ruscheweyh and Sandkuhler 2003) to induce 4–12 Hz network‐based rhythmic firing in SG. This in vitro SG rhythmic behavior possessed some similarities to patterned activity previously described for DH in vivo (EblenZajjur and Sandkuhler 1997) which persisted after spinalization suggesting localization to spinal cord circuitry (Sandkühler et al. 1995). Using this novel 4‐AP model in vitro, we demonstrated attenuation of 4–12 Hz rhythmic activity by nonspecific neuronal and glial gap junction uncouplers such as carbenoxolone or neuronal‐specific gap junction drugs such as mefloquine (Chapman et al. 2009). These data accord with the reported contribution of electrical synapses and gap junctions to the synchronization of 4‐AP‐induced neuronal network behavior in cortical regions (Maier et al. 2002).

To date, few studies have attempted to identify a spinal DH cellular mechanism for gap junction‐related changes in the context of nociception or altered spinal sensory processing. In this study, we have utilized the 4‐AP model of neonatal rat DH hyperexcitability in vitro in combination with extracellular single microelectrode recordings from within substantia gelatinosa (SG) to characterize more fully the respective contributions of specific Cx proteins, namely Cx43 which is localized to glia (Dermietzel et al. 2000) and Cx36 which is accepted as neuronal specific (Teubner et al. 2000). A quantitative reverse transcription polymerase chain reaction (RT‐qPCR) methodology was used initially to determine developmental expression of both Cx43 and Cx36 across the postnatal ages 4 days, 7 days, and 14 days in lumbar spinal cord tissue. In the electrophysiological studies, the putative contribution of Cx36 was probed using the Cx36‐specific pharmacological drug quinine (Gajda et al. 2005; Srinivas et al. 2001). In addition, we determined the contribution of Cx43 using the gap junction blocker mimetic peptide Gap 26, a novel short‐sequence synthetic peptide corresponding to residues 63–75 of Cx43 within the first extracellular loop of Cx43 (Leybaert et al. 2003), on 4‐AP‐induced DH hyperexcitability and rhythmicity. Finally, if the hypothesis that gap junction closure attenuates 4‐AP‐induced 4–12 Hz rhythmicity is correct, then conversely enhanced connectivity through gap junction could augment this 4‐AP‐induced excitation. Trimethylamine (TMA) increases the efficacy of gap junction coupling possibly through alkalization (Nassiri‐Asl et al. 2008) and is considered as a nonselective gap junction “opener”. TMA was used to test the effects of pharmacological enhancement of gap junction‐mediated network connectivity on 4‐AP‐induced activity in SG in vitro.

The overall aims of the study were firstly, to establish a causal link between an established form of rhythmic activity in SG and specific Cx subtypes, namely Cx36 and Cx43, that are known to be expressed in the spinal cord, and secondly, to demonstrate the potential for active bidirectional modulation of gap junctions channels in SG.

## Methods

### RT‐qPCR

Male Wistar rats across three postnatal age groups 4 days, 7 days, and 14 days were terminally overdosed by inhalation of isoflurane (Merial, Essex, UK). Extracted segments of L3–L6 lumbar spinal cord were stored at −80°C. RNA was extracted from L3 to L6 of the lumbar spinal cord and reverse transcribed (RT) into cDNA. To detect relevant Cx gene expression, TaqMan polymerase chain reaction was then performed using customized primers for selected Cx proteins normalized to *β*‐actin (Applied Biosystems Ltd., Warrington, Cheshire, UK). cDNA samples were detected in triplicate using a BioRad iQ5 over 40 cycles. Stock MEGA‐Master mix was prepared using 30 *μ*L of Cx probe and 300‐*μ*L qPCR Master‐Mix (Promega, Madison, WI) with 18 *μ*L of MEGA‐Master mix added to 2 *μ*L of DNA sample.

### Single microelectrode extracellular recordings

All animal procedures complied with current UK legislation as defined in the Animals (Scientific Procedures) Act 1986. Spinal cords were extracted from 12‐day‐old Wistar rats terminally anesthetized with pentobarbital (40–50 mg/kg i.p.). After removal of the pia and dura mater, 350‐*μ*m transverse slices were cut from within L3–L6 using a vibratome (Leica VT1000S, Leica Microsystems, Germany). Slices were maintained at 37°C in oxygenated (95% O_2_ and 5% CO_2_) artificial cerebrospinal fluid (ACSF) containing (in mmol/L): 126 NaCl, 2.5 KCl, 1.4 NaH_2_PO_4_, 1.2 MgCl_2_, 2.4 CaCl_2_, 25 NaHC0_3_, and 11 glucose. For electrophysiology, spinal cord slices were transferred to a Perspex Haas‐type interface chamber humidified with Carbogen (95% O_2_–5% CO_2_). All control and drug‐containing solutions were perfused using a gravity perfusion system at a flow rate of 1–1.5 mL/min.

Recordings were performed using low resistance (~2–4 MΩ) ACSF‐filled glass microelectrodes micropositioned under visual guidance into SG at a depth of 5–10 *μ*m. In spinal cord slices, the SG is clearly visible with light microscopy, appearing as a distinctive translucent arc within the superficial DH easily distinguishable from deep DH laminae. Voltage waveforms (×10) were recorded with an Axoclamp 2A system (Molecular Devices, Sunnyvale, CA) and further amplified (×1000) with a Neurolog NL106 module (Digitimer, Welwyn Garden City, UK). Voltage signals were filtered using a low pass band filter setting of 40 Hz (Neurolog NL125, Digitimer) and a Humbug device (Digitimer) for 50 Hz activity. Recordings were digitized at a sampling rate of 5 kHz and captured for further offline analysis using Spike 2 software (Cambridge Electronic Design, Cambridge, UK). Power spectra were generated using 1 sec epochs and the amplitude of the peak frequency was measured to give the power of the oscillation. Power amplitude (measured as maximum peak height in spectra) and area values (calculated as the “area‐under‐the‐curve” for two cursors set at 4 and 12 Hz in spectra) were derived from an average of six consecutive 1 sec epochs recorded within the interspike interval of large amplitude field population spiking activity. The sampling rate was 5 kHz, which divided by the 8192 points in the fast Fourier transform, provides an overall resolution of 0.6 Hz. Statistical analysis of power amplitude, power area, and frequency were performed using a Student's two‐tailed *t* test for paired or in the case of Gap 26 unpaired data using raw data values. A Kolmogorov–Smirnov test indicated that the data were consistent with a normally distributed population. Data values were expressed as means ± standard error of the mean (SEM). Values of *P < *0.05 were considered statistically significant. All of the stated *n* values refer to the number of spinal slices used per experiment taken from a minimum of three animals.

### Pharmacology

Drugs were dissolved in ACSF and applied via separate gravity‐fed inlets. All experiments involving 4‐AP (Sigma‐Aldrich, Gillingham, Dorset, UK) were carried out using 25 *μ*mol/L which has been previously shown to induce stable 4–12 Hz rhythmic activity in SG (Chapman et al. 2009). Either the gap junction opener trimethylamine (50 *μ*mol/L, TMA) (Acros Organics, Thermo Fisher Scientific, Geel, Belgium) or the selective Cx36 antagonist quinine (250 *μ*mol/L) (MP Biomedicals Incorporated, Solon, OH) were then coperfused with 4‐AP. For experiments using the Gap 26 mimetic peptide (Val‐Cys‐Tyr‐Asp‐Lys‐Ser‐Phe‐Pro‐Ile‐Ser‐His‐Val‐Arg), a comparison was made between the effects of 4‐AP on control spinal cord slices preincubated in ACSF alone and slices preincubated in ACSF containing 100 *μ*mol/L Gap 26 (Alpha Diagnostic International Inc., Nottingham, UK) for at least 4 h. During recordings, slices were perfused with Gap 26 in the continued presence of 4‐AP (25 *μ*mol/L).

## Results

### Cx36 and Cx43 expression in postnatal lumbar spinal cord

**Figure 1:**
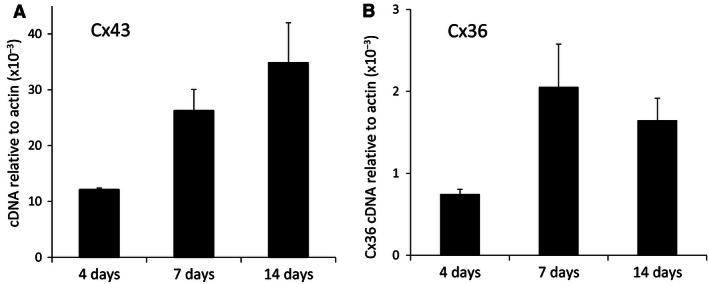
RT‐qPCR quantification of gene expression for glial Cx43 (A) and neuronal Cx36 (B) relative to actin in lumbar spinal cord across the postnatal periods P4, P7, and P14.

RT‐qPCR demonstrated the gene expression of Cx36 and Cx43 in lumbar spinal cord tissue from rats across three developmental ages: 4 days, 7 days, and 14 days postnatal. Relative to actin, the expression levels for Cx43 were measured as 12.1 ± 0.27 × 10^−3^ at P4 (*n* = 4), 26.3 ± 3.75 × 10^−3^ at P7 (*n* = 7), and 34.9 ± 7.17 × 10^−3^ at P14 (*n* = 5) (Fig. 1A) indicating an apparent rising trend in Cx43 gene expression across this postnatal period, although these differences were not statistically significant (one‐way between‐groups ANOVA test). For Cx36, the gene expression levels relative to actin (Fig. 1B) were measured as 0.8 ± 0.06 × 10^−3^ at P4 (*n* = 5), 2.1 ± 0.52 × 10^−3^ at P7 (*n* = 7), and 1.7 ± 0.27 × 10^−3^ at P14 (*n* = 4). The expression levels for Cx36 apparently increased between P4 and P7 and then decreased at P14, a one‐way between‐groups ANOVA test indicated that these differences were not statistically significant. These gene expression data infer the presence of both Cx36 and Cx43 in postnatal lumbar spinal cord including tissue age‐matched to that used for electrophysiological studies.

### 4‐AP‐induced rhythmic network activity in SG

**Figure 2:**
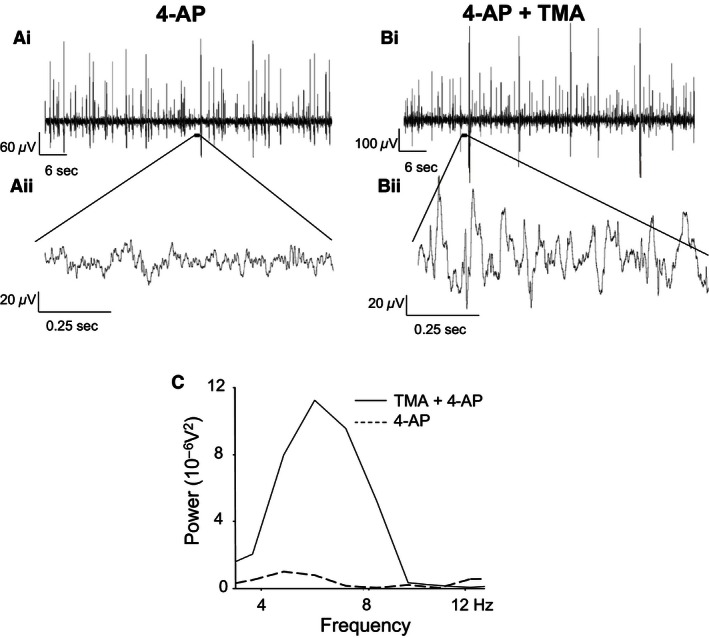
The effects of trimethylamine on 4‐AP‐induced network‐based activity in SG. (Ai) Trace recording during perfusion of 4‐AP, (Aii) 1 sec trace from within an interspike interval, (Bi) Trace recording during perfusion of 4‐AP and TMA, (Bii) 1 sec trace from within an interspike interval during TMA. (C) power spectrum of 4–12 Hz range activity in 4‐AP and coapplication of TMA and 4‐AP.

**Figure 3:**
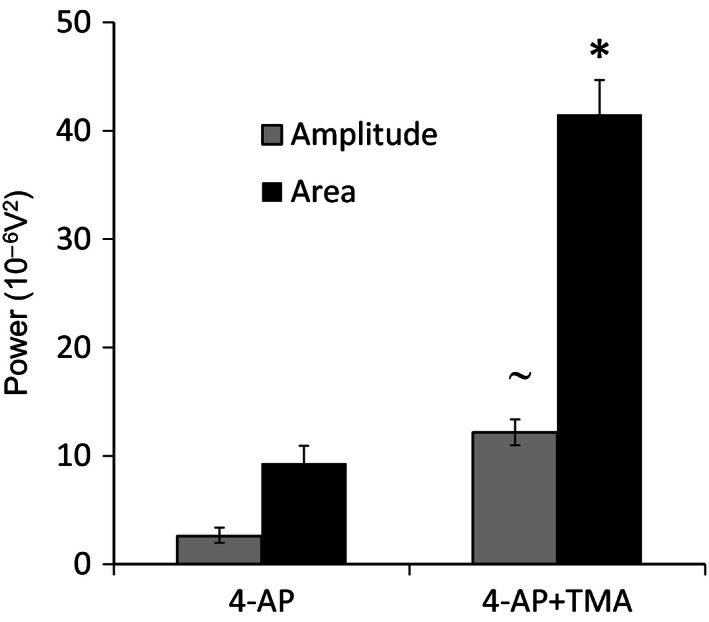
Quantification of power spectral data for effects of TMA on 4‐AP‐induced 4–12 Hz rhythmic activity in SG. Compared to 4‐AP alone, coapplication of TMA significantly increases power amplitude and power area parameters of 4–12 Hz rhythmic activity. (*P < 0.05, comparing 4‐AP and 4‐AP plus TMA for area; ^˜^ *P *< 0.05, comparing 4‐AP and 4‐AP plus TMA for amplitude).

Prolonged (30–45 min) superfusion of 4‐AP (25 *μ*mol/L) induced a typical and previously reported (Chapman et al. 2009) pattern of increased population spike and 4–12 Hz rhythmic activity within SG (Figs. 2, 3). The 4–12 Hz 4‐AP‐induced activity is characterized by rhythmic low‐amplitude voltage oscillations (*<*20 *μ*V) arising from interspike baseline activity and possesses a typical dominant mean peak frequency of 8.0 ± 0.1 Hz (Fig. 2A). This interspike 4–12 Hz rhythmic activity is distinct from the large amplitude population spikes that are also induced by 4‐AP, but do not possess a rhythmic profile (Fig. 2A). The quantified parameters of peak power amplitude and the power area of drug‐induced 4–12 Hz rhythmic activity was significantly different from low‐level baseline activity recorded in control drug‐free ACSF (*P < *0.05) (Fig. 3).

### Enhancing network activity within SG through pharmacologically increased gap junctional coupling

To assess whether an increase in intercellular gap junctional communication would affect 4‐AP‐induced 4–12 Hz network activity in SG, the gap junction “opener” TMA (50 *μ*mol/L) was utilized. Rhythmic activity was induced and recorded initially under conditions of 4‐AP (25 *μ*mol/L) alone and then in the presence of both 4‐AP and TMA (*n* = 6, 45 min). Comparing ACSF and 4‐AP, the mean power amplitude (expressed as 10^−6^V^2^) rose from a baseline value of 0.81 ± 0.46 to a value of 2.59 ± 1.5 (*P* < 0.05). The corresponding baseline value for power area was 2.48 ± 1.25 in ACSF which increased to 9.32 ± 2.5 after exposure to 4‐AP (*P* < 0.05). Subsequent coapplication of TMA and 4‐AP further increased both quantified power amplitude and power area parameters of 4–12 Hz activity. Figure 2 presents a representative recording from a single spinal cord slice showing the typical augmentation of 4–12 Hz activity by TMA (Fig 2A and B). The corresponding power spectrum for this exemplar recording (Fig 2C) shows the characteristic enhancement of power amplitude and power area induced by coapplication of 4‐AP and TMA. Figure 3 provides a comparison of quantified data (*n* = 6) for power amplitude and area in the presence of 4‐AP compared to coapplied 4‐AP and TMA. The power amplitude value rose from 2.59 ± 1.5 in 4‐AP alone to 12.17 ± 0.75 (370% increase) in TMA and 4‐AP (Fig. 3). The power area rose from 9.32 ± 2.5 in 4‐AP to 41.48 ± 0.75 (345% increase) in TMA and 4‐AP (Fig. 3). The increases in power amplitude and power area within the 4–12 Hz range following coapplication of 4‐AP and TMA were statistically significant (*P < *0.05). When tested against control baseline activity recorded in drug‐free ACSF, TMA did not by itself evoke any measurable form of rhythmic activity. In the presence of TMA with 4‐AP, the dominant activity profile was not significantly altered and remained within the 4–12 Hz range (6.00 ± 0.94 Hz in 4‐AP alone and 5.03 ± 1.24 Hz with TMA and 4‐AP, *P *> 0.05). These data indicate that while TMA cannot alone induce 4–12 Hz rhythmic activity, it can act to enhance this form of established ongoing 4‐AP‐induced activity.

### 4‐AP‐induced rhythmic activity is partly dependent on gap junctions that express Cx36 and Cx45

**Figure 4:**
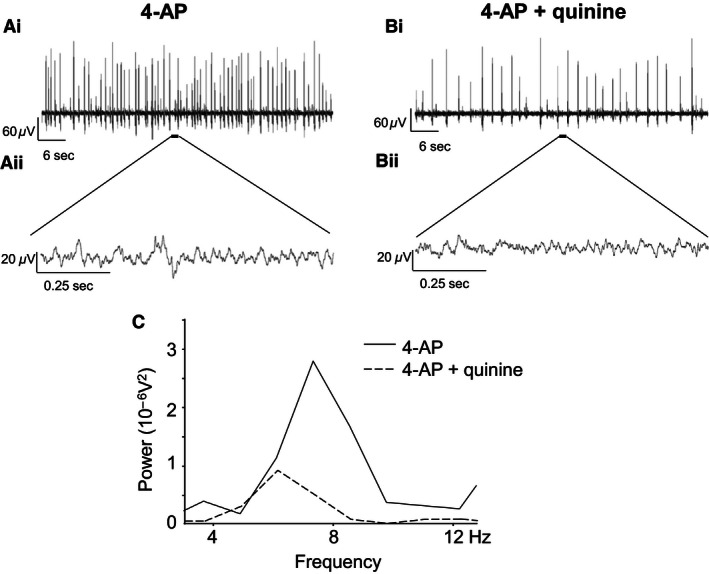
Effects of quinine on 4–12 Hz rhythmic network activity within SG of rats aged 10–14 days. (Ai) Typical recording from a single spinal cord slice showing increased excitability during superfusion of 4‐AP (25 *μ*mol/L), (Aii) 1 sec trace of activity from within an interspike interval in 4‐AP. (Bi) Trace recording after a 45‐min coapplication of 4‐AP and quinine, (Bii) 1 sec epoch of activity from within an interspike interval in 4‐AP and quinine. (C) Power spectrum of 4–12 Hz activity during 4‐AP (solid line) and 4‐AP and quinine (dashed line).

The Cx36‐specific neuronal gap junction uncoupler quinine (250 *μ*mol/L) was utilized to assess the reliance of 4‐AP‐induced 4–12 Hz rhythmic activity on neuronal gap junctions expressing this Cx subtype. After establishment of 4–12 Hz rhythmic activity by 4‐AP (25 *μ*mol/L), quinine and 4‐AP were subsequently coapplied (45 min). Quinine significantly reduced 4‐AP‐induced 4–12 Hz rhythmic activity without affecting the dominant peak frequency (7.59 ± 0.66 Hz in 4‐AP alone 7.81 ± 0.60 Hz with quinine and 4‐AP, *P* > 0.05). A typical example of this for activity recorded from a single spinal cord slice is illustrated in Figure 4.

**Figure 5:**
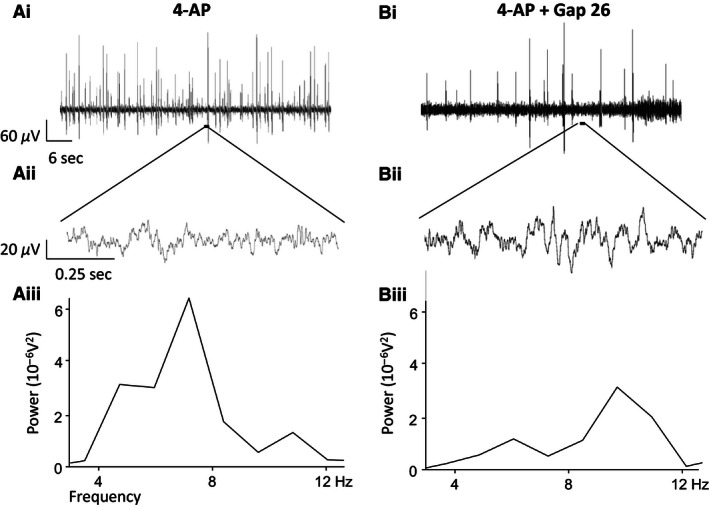
The effects of Gap 26 on generating 4‐AP‐induced network‐based activity in SG. (Ai) Trace from within SG from a control slice, untreated with Gap 26 during perfusion of 4‐AP, (Aii) Example trace within an interspike interval from a control slice, (Aiii) Power spectral analysis of the 4–12 Hz activity in 4‐AP. (Bi) Example trace from SG during perfusion of 4‐AP and Gap 26 from a slice which has been incubated in Gap 26, (Bii) Example trace within an interspike interval from a Gap 26‐incubated slice during coapplication of 4‐AP and Gap 26, (Biii) Power spectral analysis of the 4–12 Hz activity in Gap 26 and 4‐AP following incubation in Gap 26.

To test the direct involvement of astroglial gap junctions expressing Cx43, the highly specific mimetic peptide Gap 26 was utilized. A comparison was made between the level of activity induced within SG in spinal cord slices preincubated in control ACSF and then exposed to 4‐AP (45 min., 25 *μ*mol/L) versus spinal cord slices that were preincubated with Gap 26 (4 h, 100 *μ*mol/L) prior to coapplication of 4‐AP and Gap 26. Figure 5 presents typical recordings from within SG in two separate spinal cord slices obtained under these two different pharmacological conditions. While 4‐AP continued to induce a dominant 4–12 Hz rhythmic activity profile in control (Figure 5 Ai and Aii) and Gap 26‐treated slices (Fig. 5B), the level of activity was compromised by Gap 26.

**Figure 6:**
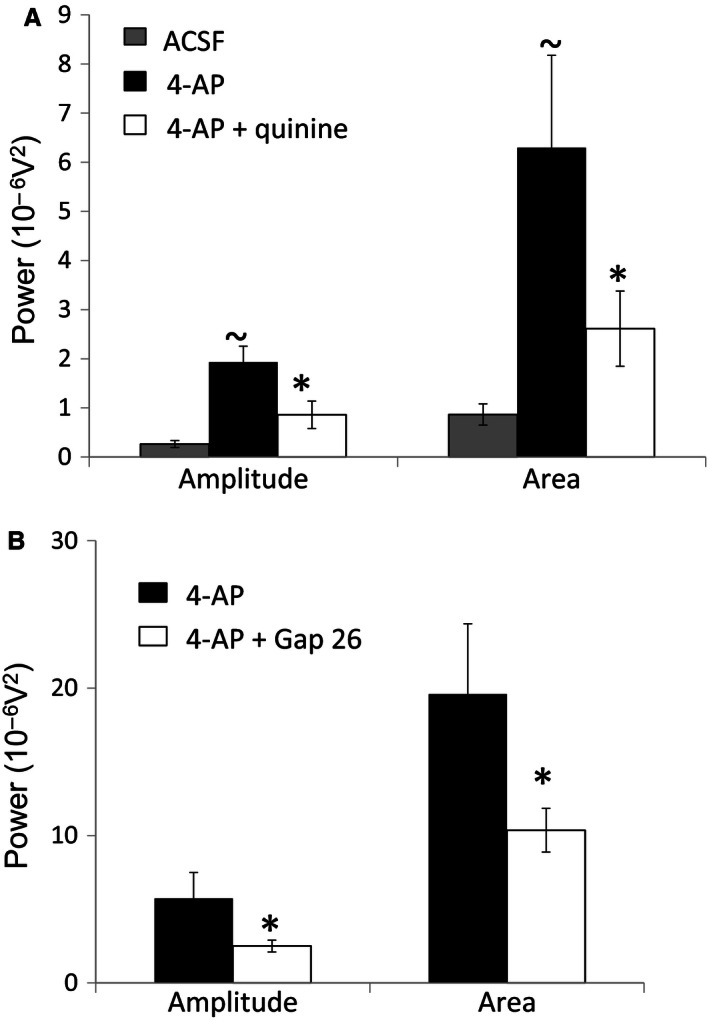
Quantification of power spectral data for the effects of quinine, Gap 26, and TMA on 4‐AP‐induced 4–12 Hz rhythmic activity in SG. (A) The attenuating effect of quinine on 4‐AP evoked activity, quantified as power amplitude and power area. Compared to ACSF alone, 4‐AP enhances activity. 4‐AP‐induced activity levels are diminished by quinine. (~*P *< 0.05, comparing 4‐AP to drug‐free ACSF; *P < 0.05, comparing 4‐AP plus quinine to 4‐AP alone for both amplitude and area). (B) Comparison of 4‐AP‐induced activity between control slices which were not preincubated in Gap 26 and spinal slices preincubated in Gap 26. Activity levels are diminished by Gap 26. (*P < 0.05, comparing 4‐AP plus Gap 26 to 4‐AP alone for both amplitude and area).

Quantified data for the effects of quinine and Gap 26 on 4–12 Hz activity are summarized in Figure 6. During cosuperfusion of quinine, power amplitude was reduced from a value of 1.93 ± 0.33 in 4‐AP alone to 0.86 ± 0.28 when quinine was coapplied (*P < *0.05, *n* = 5) (Fig. 6A). Similarly, the quantified power area value was reduced from 6.29 ± 1.88 in 4‐AP to 2.61 ± 0.77 during coapplication of quinine (*P < *0.05, *n* = 5) (Fig. 6A). These data represent percentage reduction values of 55% and 59% for power amplitude and area, respectively, by quinine and indicate that Cx36‐expressing neuronal gap junctions are necessary for the full manifestation of 4‐AP‐induced 4–12 Hz rhythmic activity in SG. For Gap 26, the mean power amplitude was 5.7 ± 1.76 and the power area was 19.6 ± 4.77 in control slices exposed only to 4‐AP (*n* = 12) (Fig. 6B). In slices preincubated with Gap 26 and then exposed to Gap 26 and 4‐AP (*n* = 19), the mean power amplitude was 2.51 ± 0.40 and power area was 10.40 ± 1.48 (Fig. 6B). These values represent a 56% reduction in power amplitude which is similar to the reduction caused by quinine and 47% for power area which is just slightly smaller than the effect of quinine on the same parameter. Statistical comparison of the magnitude of the differences in mean values for power amplitude and power area between the two groups, Gap 26‐treated versus control untreated spinal cord slices, showed a significant reduction (*P < *0.05) for both parameters under the condition of Gap 26 preincubation. These data indicate that Cx43‐expressing astrocytic gap junctions are necessary for the full manifestation of 4‐AP‐induced 4–12 Hz rhythmic activity in SG.

## Discussion

Gene expression for both Cx36 and Cx43 in immature rat spinal cord over the developmental age range of 4–14 days postnatal was revealed using RT‐qPCR. Some clear trends in gene expression levels emerged for these two Cx protein subtypes. Cx43 levels apparently increased across the three age groups tested, P4, P7, and P14, while the levels of Cx36 initially increased and then decreased. In the CNS, Cx43 is characteristic of astrocytes, meningeal, and ependymal cells and in adult rat spinal cord specifically, Cx43 is distributed throughout the dorsal and ventral gray matter with the highest levels localized to DH SG (Ochalski et al. 1997). Cx43 was immunohistochemically localized to an asymmetrically labeled gap junctions between astrocytic processes and oligodendrocytes, potentially creating an extensive gap junction‐coupled syncytium for cellular communication. In contrast, Cx36 is principally associated with neuronal gap junctions distributed throughout the adult CNS (Condorelli et al. 2000) including the gray matter of the spinal cord (Rash et al. 2000), thereby potentially providing a structural network for electrical or metabolic integration between neurons. In mouse lumbar DH, Cx36 has been functionally linked to inhibitory interneurons and is colocalized with the glycinergic interneuronal marker GlyT‐2 but not the GABAergic marker GAD67 (Nakamura et al. 2015).

In the continued presence of 4‐AP, the spinal SG in vitro manifests 4–12 Hz rhythmic activity (Chapman et al. 2009). The precise mechanistic origin of this rhythmic activity is unknown but as a K^+^ channel blocker with selectivity toward A‐currents, 4‐AP will likely exert both pre‐ and postsynaptic excitatory effects. In rat DH in vitro, 4‐AP excitation is apparently initiated in the medial superficial DH with subsequent wider propagation across the DH (Ruscheweyh and Sandkuhler 2005). In this 4‐AP model of 4–12 Hz rhythmic activity, contrasting pharmacological strategies that either enhanced or impaired gap junction conductance revealed a role for Cx‐containing electrical synapses, especially Cx36 and Cx43, in the generation and maintenance of synchronous firing across a functional syncytium within SG. In the first instance, the weak base drug TMA which enhances gap junction connectivity nonselectively by a process of intracellular alkalization was coapplied with 4‐AP (Pais et al. 2003). TMA has well‐established proepileptic properties and enhances 4‐AP‐induced epileptiform activity in cortex (Medina‐Ceja and Ventura‐Mejia 2010). Interestingly, TMA alone did not induce excitatory activity within SG but had a significant and strong enhancing effect on 4‐AP‐induced 4–12 Hz activity. This was quantified as an increase in the parameters of power amplitude and power area derived from fast Fourier spectra analysis. These data suggest the superimposition of an enhanced level of 4‐AP‐induced interneuronal synchronous excitation via a TMA‐mediated increase of local gap junction coupling efficacy within SG.

Conversely, pharmacological disruption of gap junction conductance using nonspecific uncouplers such as carbenoxolone has been shown to reduce 4‐AP‐induced 4–12 Hz rhythmic activity (Chapman et al. 2009) in spinal DH. In this study, more specific pharmacological tools have been used to causally link Cx36 and Cx43 to expression of 4‐AP‐induced 4–12 Hz synchronous activity in SG. The antimalarial drug quinine preferentially reduces currents through gap junctions that incorporate Cx36 without substantially affecting the conductance of gap junctions that express Cx43 or other Cx subtypes (Srinivas et al. 2001). Quinine has been used to probe the role of Cx36 in cortical network synchronization and seizure activity (Medina‐Ceja and Ventura‐Mejia 2010; Gigout et al. 2006). The functional deficits reported in the Cx36 knockout mice indicate a physiological relevance for neuronal gap junctions in the CNS, particularly with respect to generation of membrane oscillations and synchronous recruitment of rhythmic firing (Hormuzdi et al. 2001; Pais et al. 2003). These data show that quinine had a pronounced and significant attenuating effect on 4‐AP‐induced 4–12 Hz activity suggesting that in SG coupling through Cx36‐expressing gap junctions is an essential contributor to this form of rhythmic activity. A similar attenuating effect on 4–12 Hz activity was found using the mimetic peptide Gap 26 which is a very specific blocker of Cx43, one of the principal Cxs localized to astrocytes. Taken together, these data support the existence of an electrically and metabolically coupled functional syncytium distributed across both the neurons and glia within immature SG that is capable of supporting rhythmic excitatory activity. The speculative extrapolation of this finding to the mature rodent spinal SG is justified on the basis of persistent expression into adulthood of Cx36 and Cx43 (Ochalski et al. 1997; Condorelli et al. 2000; Nakamura et al. 2015; Rash et al. 2000) as well as several other Cx subtypes (Chapman et al. 2013; Lee et al. 2005).

The physiological role of such a gap junction network within SG and its relevance to nociceptive processing within SG is unknown. However, the concept of gap junctions functioning simply as passive intercellular conduits is no longer tenable given the substantive evidence that gap junction channel expression and conductance are modifiable by factors such as neuropeptides, cytokines, or growth factors (Giaume et al. 2010). Furthermore, it seems clear that changes in expression or function may be linked to pathological conditions including neuropathic pain and chronic pain states (Wu et al. 2012). For example, after L5 spinal nerve ligation, astrocytic Cx43 is upregulated and suppression of spinal Cx43 expression inhibited injury‐induced mechanical hypersensitivity (Xu et al. 2014). In another study, chronic constriction injury‐induced mechanical allodynia was at least partially ameliorated by Gap 26, the Cx43‐specific blocker used in this study (Chen et al. 2014). Cx43 but not Cx36 expression is reportedly increased in models of chemotherapy‐induced peripheral neuropathy with a shift in Cx43 to a more phosphorylated state (Robinson and Dougherty 2015; Yoon et al. 2013). Altered spinal expression of Cx43 has also been characterized in models of spinal cord injury (SCI) and pain (Chen et al. 2012; Huang et al. 2012). In mice with Cx43/Cx30 deletions, thermal hyperalgesia and mechanical allodynia triggered by SCI were prevented (Chen et al. 2012). In contrast to the reportedly increased Cx43 expression under chronic pain conditions, after a peripheral nerve injury, Cx36 expression was markedly reduced, with the time course of change paralleling the emergence of tactile allodynia (Nakamura et al. 2015), and this effect could be mimicked by injection of Cx36 siRNA to knock down Cx36 expression. Such studies implicate altered communication via Cx gap junctions as a potential contributory factor to central sensitization perhaps, for example, by more widespread or increased aberrant excitation across a modified functional syncytium.

In conclusion, these electrophysiological data reveal a role for neuronal Cx36 and astrocytic Cx43 gap junctions in a form of rhythmic firing in immature SG that can be induced in vitro by 4‐AP. Further studies will be required to elucidate the functional significance of these data in the mature nociceptive DH and, in the context of acute versus chronic pain, the importance of Cx as a future analgesic drug target.

## Conflict of Interest

None declared.
